# Lung Ultrasound Assessment of Regional Distribution of Pulmonary Edema and Atelectasis in Infants with Evolving Bronchopulmonary Dysplasia

**DOI:** 10.3390/diagnostics14202341

**Published:** 2024-10-21

**Authors:** Jimikumar Patel, Barry Weinberger, Margaret Pulju, Stephanie G. Galanti, Gangajal Kasniya, Venkata Gupta, Dalibor Kurepa

**Affiliations:** 1 Division of Newborn Medicine, Department of Pediatrics, University of Mississippi Medical Center, 2500 North State Street, Jackson, MS 39216, USA; jimimd0104@gmail.com; 2 Division of Neonatal-Perinatal Medicine, Northwell Health, Cohen Children’s Medical Center, Donald and Barbara Zucker School of Medicine at Hofstra/Northwell, 269-71 76th Street, New York City, NY 11041, USA; bweinberger@northwell.edu (B.W.); mikkipulju@emory.edu (M.P.); sgalanti@northwell.edu (S.G.G.); dr.kasniya@gmail.com (G.K.); guptavmd@gmail.com (V.G.)

**Keywords:** lung ultrasound, bronchopulmonary dysplasia, regional ventilation, pulmonary edema, atelectasis

## Abstract

**Background:** Preterm infants are at risk for bronchopulmonary dysplasia (BPD) due to prolonged respiratory support. Studies have described differences in the regional distribution of lung ventilation (non-dependent (NDL) vs. dependent (DL)). The aim of this study was to use LUS to compare regional distribution of pulmonary edema and atelectasis in infants with evolving BPD. **Methods:** We prospectively performed LUS in premature infants with evolving BPD. On each side, three lung areas (NDL/anterior, lateral, and DL/posterior) were examined for the presence of pulmonary edema and atelectasis. Pulmonary edema scores were assigned based on the number of B-lines, and atelectasis scores were assigned based on the presence/absence of atelectasis. **Results:** 38 premature infants were enrolled. The NDL showed more pulmonary edema and atelectasis compared to the DL (*p* = 0.003, *p* = 0.049, respectively) and compared to the lateral lung (*p* =< 0.001, *p* = 0.004, respectively). There was no difference between the lateral and DL (*p* = 0.188, *p* = 0.156, respectively). There was no difference between the right and the left lung (*p* = 0.223, *p* = 0.656, respectively). **Conclusions:** In this cohort of preterm infants with evolving BPD, lung disease was unevenly distributed, with more pulmonary edema and atelectasis in the NDL regions compared to the DL or lateral regions.

## 1. Introduction

Bronchopulmonary dysplasia (BPD) is a complex cardiorespiratory morbidity that affects preterm infants. It signifies an important indicator for benchmarking the quality of neonatal care [1]. Despite recent advances in neonatal care, the prevalence of BPD has remained constant mainly due to improved survival of extremely low gestational age newborns [2]. Pathologically, BPD is characterized by lung injury with disrupted alveolarization, atelectasis, inflammatory cell proliferation, pulmonary edema, and vascular remodeling. Infants with BPD are at increased risk for prolonged respiratory support, pulmonary hypertension, compromised growth, respiratory-related rehospitalization, and neurodevelopmental deficits [3].

Although the diagnosis of BPD is usually based on chest X-ray and clinical findings, lung ultrasound (LUS) has gained popularity in recent years because of its portability and lack of ionizing radiation. Several important publications have supported the use of LUS for diagnosing BPD, tracking its progression, and assessing response to therapy [4,5,6,7]. In supine infants, the dependent (posterior) lung regions (DL) are relatively compressed and have lower resting volumes than the non-dependent (anterior) lung regions (NDL), requiring higher intrapleural pressure to maintain expansion. In healthy infants, the DL regions expand more on inspiration and are paradoxically better ventilated than the NDL regions [8]. However, this normal distribution of ventilation may be inverted in the pathologic setting of poor lung compliance, such as respiratory distress syndrome (RDS), because of the inability of DL regions to fully expand on inspiration [9].

Premature infants with RDS have inadequate surfactant, resulting in poor lung compliance, low lung volumes, and the maldistribution of ventilation between lung regions. The primary therapeutic goal for RDS is to optimize lung volumes using exogenous surfactant and continuous positive airway pressure (CPAP) [10,11]. However, regional and position-dependent differences in lung expansion and ventilation persist in infants with RDS, regardless of whether they are spontaneously breathing or mechanically ventilated [12,13,14,15,16,17]. Recent studies using electrical impedance tomography (EIT) have also suggested that the severity of inhomogeneity of lung expansion in infants with RDS, especially in NDL, is associated with the risk of developing BPD [14]. Nevertheless, the functional dynamics of regional lung expansion in BPD have not been previously described using bedside LUS.

The aim of this study was to characterize the regional distribution of pulmonary edema and atelectasis in spontaneously breathing infants with evolving BPD. 

## 2. Methods

We prospectively (July 2022–August 2023) studied premature infants (<32 weeks gestational age) with evolving BPD, defined as the ongoing need for invasive or non-invasive respiratory support (NIPPV, CPAP, HFNC > 2 lpm) and/or supplemental oxygen between postnatal day 28 and 36-weeks post-menstrual age. This study was approved by the Northwell Health Institutional Review Board. Informed consent was obtained from parents before enrollment. Infants with major congenital anomalies, lung anomalies, or cardiac anomalies were excluded from this study.

**Figure 1:**
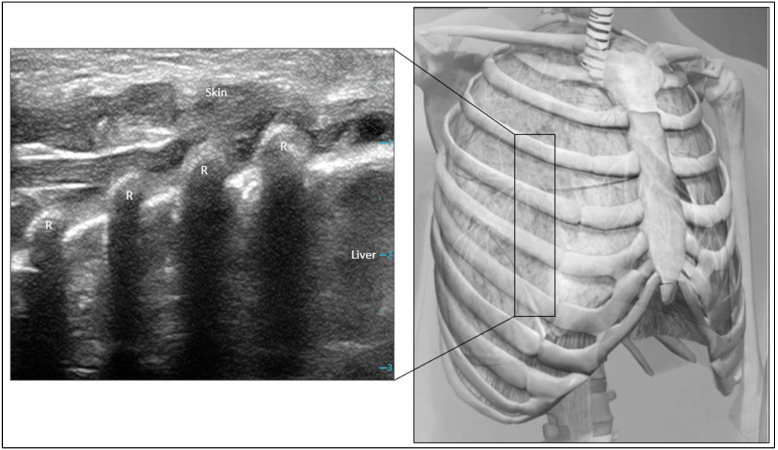
Lung ultrasound exam (**left**). Three intercostal spaces were scanned, anterior (NDL), lateral, and posterior (DL), on each side. R—rib. Image (**right**) Courtesy Visible Body.

Before each exam, infants spent ~3 h in the supine position. For each lung, three regions (NDL, lateral, and DL) were evaluated by LUS, with scans of each region covering at least four ribs and three intercostal spaces (Figure 1). First, we scanned the mid-anterior chest (NDL) bilaterally along the mid-clavicular line to include 4 ribs and 3 intercostal spaces. For the lateral region, we scanned along the mid-axillary line, and for the posterior region we scanned along the mid-scapular line (DL). During LUS exams, infants were placed on a Z-Flo^TM^ positioner that allows for individually molded nesting. To acquire posterior lung region images, we slipped a linear hockey-stick probe underneath the infant (Z-Flo is soft, easily molded, and raised from the baby’s bed) and scanned along the mid-scapular line. We started our image acquisitions on the right side anterior and the lateral and posterior regions, followed by the left side in the same sequence. We maintained this sequence for all patients, with the typical exam lasting 2–3 min. A brief video clip for all 6 areas was recorded and scored. Pulmonary edema scores (0–2) were assigned based on the number of B-lines (0 = normal lung/A-lines or <3 B-lines, 1 = ≥3 B-lines, 2 = coalescent B-lines), and atelectasis scores (0–1) were assigned based on the presence of atelectasis (0 = no atelectasis, 1 = any atelectasis). Ultrasound exams were performed with the Zonare Z One Pro machine (Mindray, Shenzhen, China) using a 14 MHz linear transducer. Ultrasound gel was pre-warmed, and the transducer was disinfected. During the exams, infants were consoled with a pacifier, and no sedation was used.

Study data were managed using REDCap electronic data capture tools hosted at Northwell Health. Continuous variables were reported as means and standard deviations, unless otherwise stated. Categorical variables were reported as frequencies and percentages. To check for normal distribution, Shapiro–Wilk tests were performed. Since all data were nonparametric, statistical analyses were carried out using two-sided Wilcoxon signed-rank tests. Ultrasound scores were compared between lung regions (NDL, lateral, and DL) and between lung sides (left and right). Any *p*-value less than 0.05 was considered statistically significant. Analyses were conducted using R (V4.2.1). All patient data are freely accessible in the REDCap database upon request.

## 3. Results

During the study period, 38 premature infants with evolving BPD were enrolled. Initially, 51 families were approached to participate in the study, 11 did not consent to participate, and two infants were excluded, one with congenital heart disease and the other with a chest deformity. LUS exams were performed at 53 ± 15.5 days of life with a mean corrected GA of 35 ± 2.2 weeks. Among the cohort, 20 patients were on bubble CPAP and 18 patients were on NC at the time of the LUS exam, with a mean FiO_2_ of 26.2 + 6.4%. Demographic and clinical characteristics are presented in Table 1. No infant had clinical deterioration during or after the LUS exam.

**Figure 2:**
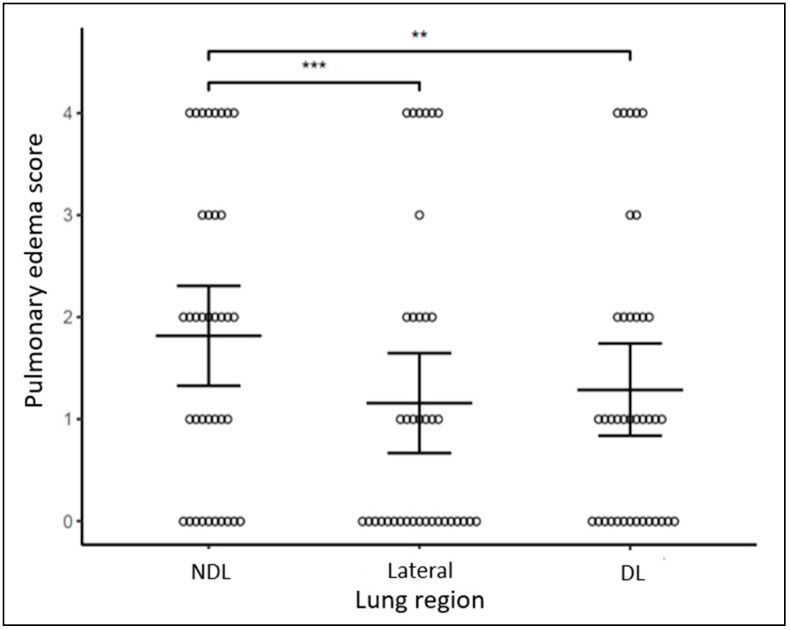
Pulmonary edema score across different lung regions. Bars represent mean estimates and 95% confidence intervals. *** *p* < 0.001, ** *p* < 0.01.

**Figure 3:**
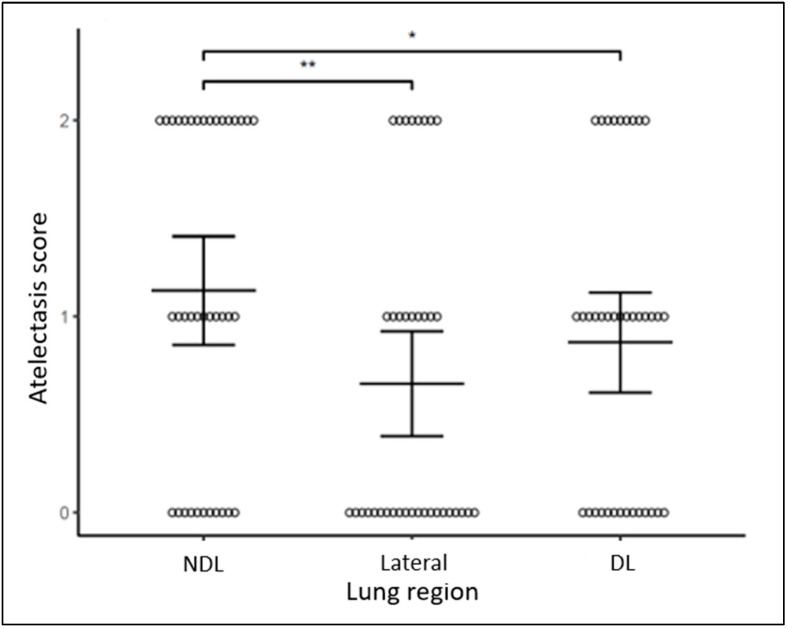
Atelectasis score across different lung regions. Bars represent mean estimates and 95% confidence intervals. ** *p* < 0.01, * *p* < 0.05.

**Figure 4:**
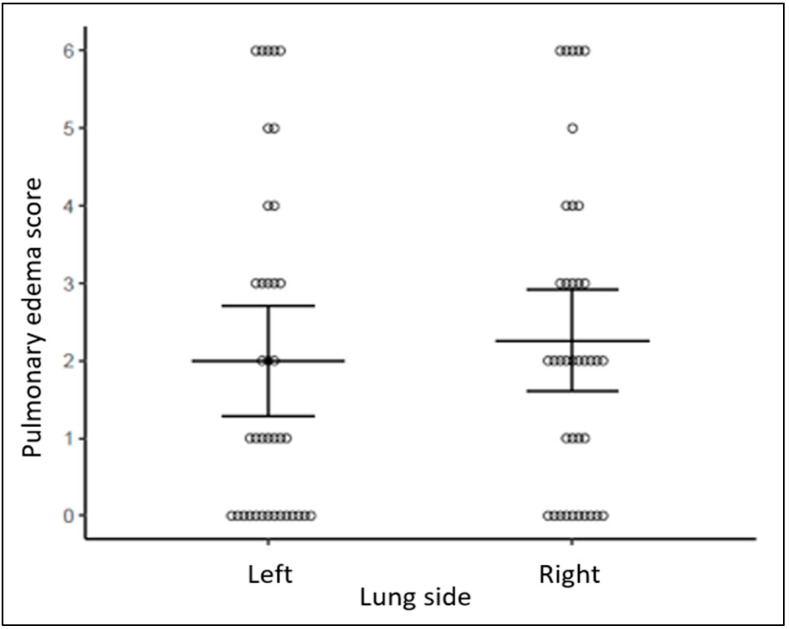
Pulmonary edema score between left and right lung side. Bars represent mean estimates and 95% confidence intervals.

**Figure 5:**
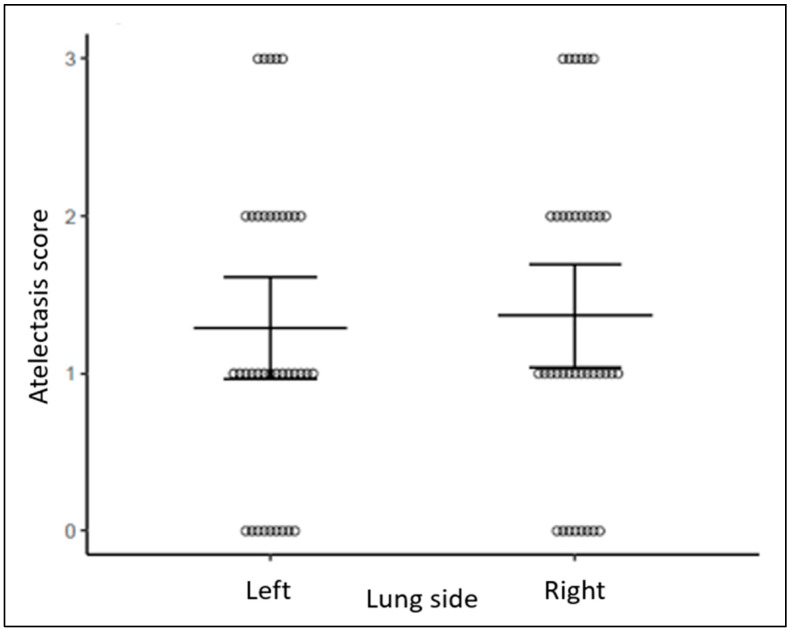
Atelectasis score between left and right lung side. Bars represent mean estimates and 95% confidence intervals.

Composite scores demonstrated more pulmonary edema and more atelectasis in NDL than DL regions bilaterally (*p* = 0.003, *p* = 0.049, respectively, Figure 2 and Figure 3). Pulmonary edema and atelectasis were also more severe in NDL than in the lateral lung regions (*p* < 0.001, *p* = 0.004, respectively). By contrast, pulmonary edema and atelectasis were not different when comparing between DL and lateral lung regions (*p* = 0.188, *p* = 0.156, respectively, Figure 2 and Figure 3) or between the right and the left lungs (*p* = 0.223, *p* = 0.656, respectively, Figure 4 and Figure 5). 

## 4. Discussion

We found that pulmonary edema and atelectasis, as detected by LUS, are more prominent in NDL regions of preterm infants, compared to the DL or lateral regions. There is no difference in the amount of pulmonary edema or atelectasis between the right and the left lung. To our knowledge, this is the first report on the regional distribution of lung changes in preterm infants with BPD using LUS. Our findings suggest that NDL regions of the lung are more vulnerable to the pathologic changes in BPD than the DL regions.

Worsened BPD in NDL regions of the lung may be explained by the pathophysiology of RDS. In a normal lung, DL regions expand more on inspiration and are paradoxically better ventilated than NDL regions [8]. However, in RDS, lung compliance is decreased due to surfactant deficiency, and DL does not expand effectively against the force of gravity. Expansion of DL in RDS may also be impaired by the pathologic accumulation of interstitial water related to the immaturity of epithelial sodium channels [18,19,20]. As a result, poorly compliant DL regions do not fully expand on inspiration in infants with RDS, and this may protect DL from injury [9]. Conversely, NDL is better expanded and is therefore disproportionately subjected to volutrauma in early RDS, leading to alveolar injury. Increased exposure of NDL to alveolar injury in RDS may explain why pulmonary edema and atelectasis, as detected by LUS, are more prominent in NDL regions in infants with established BPD. Consistent with this concept, the risk of progression to BPD is determined, in part, by the severity of inhomogeneity of lung expansion in NDL in infants with RDS [14].

Thus, our findings suggest that chronic lung disease in premature infants with RDS evolves in two distinct phases. During the first phase, when respiratory support is initiated, it is likely that the DL regions are disproportionately injured. This early pattern of injury would be compatible with that demonstrated by Louis et al., who reported that DL regions are most severely affected in infants with gestational age ≥ 29 weeks in the first day of life [21]. The second phase of lung injury occurs after several weeks of respiratory support. When we examined infants at a mean postnatal age of 53 days, they had been on respiratory support for several weeks. During that time, NDL was preferentially ventilated, exposed to higher positive pressures and greater tidal volumes. Our findings suggest that NDL is more affected than DL in this late phase (“evolving” BPD) and that positioning to protect NDL could be protective. 

The idea of a biphasic evolution of disease in BPD is supported by a recent study by Hoshino et al., which demonstrated that both gravity and time strongly affect air distribution patterns in the lungs of preterm infants with RDS who are progressing toward BPD [22]. They found that DL regions have more atelectasis on DOL 7, 14, and 21 but not on DOL 28. This suggests that NDL is well ventilated during the first 3–4 weeks. During this time, surfactant generation, respiratory muscle function, and epithelial Na channel function in the lung improve. This exposes NDL to increasing volutrauma and barotrauma, which causes worsening pulmonary edema by day 28. Most studies using LUS to evaluate the evolution of disease in BPD have focused on premature infants ≤ 28 days of age with RDS and/or TTN, and there is a paucity of such studies evaluating the second phase of injury in older infants with evolving or established BPD. 

Real-time assessment of regional lung edema and atelectasis by LUS may offer clinicians the opportunity to intervene. Gravity-induced atelectasis in preterm infants with RDS is greatest in the first 21 days after birth [22]. It is possible that regularly adjusting the position of these infants can ameliorate injury to NDL regions and slow the progression to BPD. Frequent change in infant position may minimize lung overdistention and disparities in regional volume distribution [23,24]. The improvement in regional distribution of aeration peaks at two hours after each change in body position and is sustained for two hours [25].

Due to its safety profile, portability, and ease of use, LUS has become an important tool in the respiratory management of preterm infants. It has been useful in several diagnostic and functional applications such as the evaluation of oxygenation and need for surfactant, weaning from respiratory support, prediction of BPD, and therapeutic guidance [26,27] Regional lung volumes can be assessed using methods other than LUS, but these methods are generally more invasive and/or not available in most clinical settings. Gas dilution and plethysmography techniques are limited to the research realm due to their logistical challenges and lack of robust reference data [28]. Chest X-ray cannot be used to assess lung volumes in preterm neonates [29]. Chest CT scan can be used for functional lung volume assessment, but its use is limited due to the significant radiation exposure that is required [30]. Similarly, functional lung MRI can assess ventilation, but the long procedure time and the need for infant sedation discourage its use [31,32]. Another technique, EIT, has recently became widespread in lung research for the rapid assessment of lung volumes and ventilation [12,13,14,15,23,24,33,34,35]. However, clinical application of EIT remains limited because of the complexity of the equipment and limited availability. Several studies in preterm animals have shown that LUS yields real-time data on lung volumes and regional aeration that are comparable to those obtained by EIT [36,37,38].

A strength of our study is that the LUS exams were performed by one sonographer, which minimizes the variability of image acquisition. In addition, the sonographer was masked to the infants’ clinical status, minimizing the risk of bias. This was a single-center study, which ensured that the approaches to respiratory management and positioning of preterm infants were uniform between subjects. A potential weakness of this study is that the validity of our findings is dependent on the ability of LUS to detect small changes in lung volumes in preterm infants. Although LUS findings correlate closely with EIT in animal studies, it is not yet fully validated for application in the clinical setting. The generalizability and significance of our findings may also be limited by the small sample size, and further studies of larger cohorts will be needed to validate these results in future research.

## 5. Conclusions

In this cohort of preterm infants with evolving BPD, lung disease was unevenly distributed, with more pulmonary edema and atelectasis in the NDL regions compared to the DL or lateral regions. LUS is a feasible and effective tool to assess these changes in real time at the bedside. Further studies are warranted to assess the impact of changes in body position on the incidence and severity of BPD and other long-term pulmonary outcomes for preterm infants.

## Figures and Tables

**Table 1:** Demographic and clinical characteristics.

Infant Characteristics	Cohort (N = 38) ^
Gestational age (weeks)	27.02 ± 1.73
Birth weight (g)	898.16 ± 304.28
Sex (male)	18 (47)
Small for gestational age (*n*)	12 (32)
Antenatal steroids (*n*)	28 (74)
Chorioamnionitis (*n*)	8 (21)
Delivery type (Cesarean section)	29 (76)
Apgar 5 min	8 (6–8)
Day of life at LUS exam (days)	53 ± 15.5
Gestational age at LUS exam (weeks)	35 ± 2.2
FiO_2_ (%)	26.2 ± 6.4
Respiratory rate (bpm)	56.4 ± 7.3
CPAP (*n*)	20 (53)
CPAP level (mm Hg)	5.6 ± 1.6
NC flow (lpm)	2.3 ± 0.5

^ Continuous variables: Mean ± SD, Categorical variables: Count (%).

## Data Availability

The data presented in this study are available in RedCap database on request from the corresponding author due to privacy issues.
